# Hand-washing practices at critical times in relation to childhood illnesses and under-nutrition among caregivers in Rongai sub-county, Kenya

**DOI:** 10.4314/ahs.v26i1.7

**Published:** 2026-03

**Authors:** Sharon Chepng'eno Kemboi, Dorothy Mituki Mungiria, Rose Chepchirchir Ramkat, Maureen Jepkorir Cheserek

**Affiliations:** 1 Department of Human Nutrition, Faculty of Health Science, Egerton University, Rift Valley, Kenya; 2 Department of Biological Sciences, Moi University, Eldoret, Rift Valley, Kenya

**Keywords:** Hand-washing, childhood illnesses, nutritional outcomes

## Abstract

**Background:**

Poor food handling creates a pathway for childhood illnesses leading to under-nutrition.

**Objective:**

To assess hygiene practices of caregivers of young children at critical points in relation to childhood infections and under-nutrition in Rongai sub-county, Nakuru, Kenya.

**Methods:**

A cross-sectional study was conducted among 384 caregivers of children aged 6-23 months. A structured questionnaire was used to assess hygiene practices, history of infections and anthropometric measurements of children to determine z-scores.

**Results:**

Majority of caregivers were aware of some critical hand-washing times; before handling food (82.6%), before feeding the child (71%), after handling raw food (60.9%) and garbage (68.7%). Washing hands under running water [AOR=0.078, 95% C.I; (0.008±0.795)], before feeding the child [AOR=0.175 95% C.I; (0.043±0.709)], and after handling garbage [AOR= 0.273 95% C.I; (0.089±.831)] reduced chances of experiencing diarrhoea among children aged 6-11 months. Further, underweight was positively associated (P<0.05) with parasitic infections [AOR=3.123 95% C.I; (1.047±9.317)] and having trouble in breathing [AOR=6.212 95% C.I;(1.596±24.184)].

**Conclusion:**

Adequate hygiene practices among caregivers during critical times were associated with reduced chances of childhood illnesses in children aged 6-11 months. Behaviour change communication and education of caregivers about critical hand washing times during complementary feeding period are needed for improved child nutrition outcomes.

## Introduction

The first one thousand days of life is considered a significant period for growth and development. The 6-23 month age period is particularly an important stage as it is the complementary feeding period. Inadequacies in complementary feeding are a major contributor to childhood under-nutrition, increased risk for morbidity and mortality, delayed motor development, impaired cognitive development, and reduced educational attainments and social capacities^1^,^2^. For example, poor handling of foods during this period creates a pathway for infections and other childhood illnesses.

Poor water, sanitation and hygiene (WASH) practices contribute to childhood infections such as diarrhoea, which is the second leading cause of death among children under five^3^,^4^,^45^,^46^. A high population still lack access to improved WASH despite great gains in WASH improvement during the millennial development goals era^5^. In Kenya, households with access to drinking water from improved sources has improved from 63% to 71%^6^,^7^. However, more than half of the households do not treat drinking water, which creates a risk of diarrhoeal and parasitic infections^7^.

There has been an increase in diarrhoea cases from 49% to 58% among young children^7^. Prevalence is highest among children aged 6-11 months and 12- 23 months at 27% and 24% respectively. In Nakuru county prevalence of diarrhoea among children is 11.7%^7^. This could be attributed to the low level of hygiene observed during complementary feeding. Hygiene practices during preparation, feeding and storage of complementary feeds are important since contaminated food form part of the major route of transmission of diarrhoea among infants^8^. Currently in Nakuru County, stunting level is 27.6% among children aged 6-23 months^7^. Severe infections can lead to under-nutrition, which may have long-term consequences on linear growth especially when food available is insufficient to hasten recovery from the infection^9^. In Maternal and Child Nutrition Lancet Series, hygiene and sanitation interventions reduced diarrhoeal incidences by 30% translating to a reduction of child stunting by 2.4% at the age of 36 months^10^. Unsanitary conditions and repeated diarrhoea have been linked with stunting which is still a public health concern^11^,^12^,^47^. These show that improving hygiene practices will reduce the incidences of childhood infections and subsequently under-nutrition. Therefore, the objective of this study was to assess the mother/caregiver hygiene practices during the critical times and its association with childhood infections/illnesses and subsequently under-nutrition in Rongai sub-county.

## Methods

### Study area

This study was conducted in Rongai Sub-County of Nakuru County in Kenya. Rongai Sub-County is characterised by two agro-ecological zones; low agricultural potential areas (altitude of 1520 to 1890m with average rainfall of 760mm annually) and high agricultural potential areas (altitude of 1800 to 2400m with average rainfall of up to 1270 mm annually)^13^,^14^.

### Research design

An analytical cross-sectional research design was conducted in the lean season of 2016 from March to April. This design allowed for quantification of the relationship between variables. Rainfall was at its lowest with a scarcity in food supply during this season. This survey was part of a larger study which aimed at determining the relationship between agro-biodiversity, infant and young child feeding practices and diet diversity of women and children in Rongai Sub-County^13^,^14^.

### Sampling Procedure

Purposive sampling was used to select two divisions; Kampi Ya Moto from low agricultural potential areas and Menengai from high agricultural potential areas^13^,^14^. The target population was mother/caregivers with children aged 6-23 months who had started complementary feeding. A sample size of 384 mother/caregiver-child pairs was determined using Fischer's formula^15^ and was randomly selected to participate in the study. Proportionate sampling technique was employed to determine the sample proportion of participants from Kampi Ya Moto (n=158) and Menengai (n=226) divisions. The study participants were recruited at household level with the help of community health volunteers and village elders.

### Data collection tools and procedures

#### Child health characteristics and hygiene practices

A structured questionnaire was used to collect data on socio-demographic characteristics, child infection/illness history in the previous two weeks and mother/caregivers hygiene practices. The illnesses history assessed included; fever, cough/cold, fast breathing, diarrhoea, parasites diagnosis and treatment of parasites. The responses for these illnesses were “Yes” or “No” in the presence or absence of illnesses respectively. The hygiene practices investigated included water treatment and storage practices, hand washing procedure, key moments for washing hands and how to avoid food contamination. A composite indicator of adequate and inadequate practices was then developed. Adequate hygiene practices were scored as 1 while inadequate practices were scored as 0. The sum of the scores was then computed to yield a maximum score of 13 and cut-off points then formulated: scores of 0-6 was categorised as inadequate practices while scores of 7-13 categorised as adequate practices.

#### Child Anthropometry

Weights and lengths of the children were measured using Seca body weighing scales and Seca length mats respectively following procedures outlined by Cogill (2013). Child age was verified from the Mother and Child Health booklet. The weight, height and age data collected were used to compute the three z-score indices for height-for-age, weight-for-height and weight-for-age to determine stunting, underweight and wasting respectively. These indices were based on growth standards developed by WHO (2006)^16^.

### Data Analysis

Data was analysed using Statistical Package for Social Scientists (SPSS), version 20.0^17^. Data on mother/caregiver hygiene practices and child health characteristics was analysed using chi-square tests by comparing children aged 6-11 months and 12-23 months categories and across the two agro-ecological zones (low and high agricultural potential areas). Binary logistic regression was used to determine the association of mother/caregiver hygiene practices, child health history and child Z-scorewith P<0.05 considered statistically significant at 95% confidence interval.

## Ethical Considerations

Ethical clearance and research permit were obtained from Egerton University Ethical Clearance Board and National Council of Science and Technology (NACOSTI) respectively. Permission was sought from authorities at Sub County, location and sub location levels of Rongai Sub-County before data collection. The purpose of the study was explained to the participants and informed consent, both written and verbal where necessary, was obtained by the researcher before any data collection started.

## Results

### Socio-demographic characteristics of mothers/caregivers and children aged 6-23 months in Rongai sub-county

Socio-demographic characteristics of mothers/caregivers and children of Rongai sub-county are described in Table 1. The average age of mothers/caregivers was 27.40±7.67 years (children aged 6-11 months category) and 28.80±7.55 years (children aged 6-11 months category). The average age of children who participated in the study was 8.97±1.64 months (children aged 6-11 months category) and 18.29±3.77 months (children aged 12-23 months category). Overall, there were more male children in this study (children aged 6-11 months = 50.9%; children aged 12-23 months = 51.9%). Most of the mothers/caregivers had low education level (children aged 6-11 months = 50.9%; children aged 12-23 months = 54.8%). There were no observed significant differences in socio-demographic factors across the agro-ecological zones.

**Table 1:** Socio-demographic characteristics of mothers/caregivers and children aged 6-23 months in low and high agricultural potential area of Rongai Sub-County

	Children aged 6 – 11 months							Children aged 12 - 23 months						

	Total		Low potential area		High potential area		P value	Total		Low potential area		High potential area		P value

	n	%	n	%	n	%		n	%	n	%	n	%	
**Mother/caregiver age in years^#^**	27.40±7.67		27.70±8.65		27.16±6.88		0.712	28.80±7.55		27.95±6.62		29.37±8.08		0.136
**Child age in months^#^**	8.97±1.64		8.80±1.68		9.05±1.61		0.593	18.29±3.77		18.21±3.78		18.35±3.77		0.777
**Child gender**														
Male	58	50.9	24	48	34	53.1	0.587	140	51.9	51	47.2	89	54.9	0.214
Female	56	49.1	26	52	30	46.9		130	48.1	57	52.8	73	45.1	
**Mother/caregiver education level**														
Low	58	50.9	25	50	33	51.6	0.868	148	54.8	54	50	94	58	0.194
High	56	49.1	25	50	31	48.4		122	45.2	54	50	68	42	
**Marital status**														
Married	85	74.6	32	64	53	82.8	0.068	209	77.4	82	75.9	127	78.4	0.442
Single	27	23.7	17	34	10	15.6		52	19.3	23	21.3	29	17.9	
Widowed	2	1.8	1	2	1	1.6		6	2.2	1	0.9	5	3.1	

# Data are mean ± standard deviations; Low education level = received no education and primary school education; High education level = secondary school education and tertiary level

### Source of water, storage and treatment practices among mothers/caregivers in Rongai sub-county

Table 2 presents water sources, storage and treatment practices among households in Rongai sub-county. The most common sources of water were borehole, (children aged 6-11 months = 20.2%; children aged 12-23 months = 23.3%) and surface water (children aged 6-11 months = 21.1%; children aged 12-23 months = 20%) which were significantly different (P<0.05) between low potential and high potential areas. Over half of the mothers/caregivers used soap and water to clean water container (children aged 6-11 months = 54.4%; children aged 12-23 months = 59.5%), with a significant difference (P<0.05) among mothers/caregivers of children aged 12-23 months from low and high potential areas.

**Table 2:** Source of water, storage and treatment practices among mothers/caregivers in Rongai sub-county across two child age categories and agro-ecological zones

	Children aged 6 – 11 months							Children aged 12 - 23 months						

	Total		Low potential area	High potential area			P value	Total		Low potential area		High potential area		P value

	n	%	n	%	n	%		n	%	n	%	n	%	
**Main source of water**														
Piped water into dwelling	18	15.8	5	10	13	20.3	0.01*	32	11.9	18	16.7	14	8.6	0.001*
Surface water	24	21.1	24	48	0	0		54	20	50	46.3	4	2.5	
Borehole	23	20.2	8	16	15	23.4		63	23.3	19	17.6	44	27.2	
Public pipe	11	9.6	1	2.0	10	15.6		40	14.8	4	3.7	36	22.2	
Rainwater	14	12.3	7	14	7	10.9		29	10.7	9	8.3	20	12.3	
**Treatment of water container**														
No treatment	40	35.1	22	44	18	28.1	0.11	87	32.3	32	29.6	55	34.2	0.044*
Use water and soap	62	54.4	23	46	39	60.9		16 0	59.5	65	60.2	95	59	
Others^††^	12	10.5	5	10	7	10.9		18	6.7	11	10.2	7	4.3	
**Water storage**														
Clean container	24	21.1	15	30	9	14.1	0.067	41	15.2	24	22.2	17	10.5	0.043*
Covered container	30	26.3	14	28	16	25		61	22.6	20	18.5	41	26.3	
Clean and covered container	60	52.6	21	42	39	60.9		16 3	60.4	63	58.3	10 0	61.7	
Don't know														
**Water treatment**(Yes)	65	57	31	62	34	53.1	0.342	14 6	54.1	54	50	92	56.8	0.273
**Water treatment method^#^**														
Boiling	63	55.3	30	60	33	51.6	0.369	126	46.7	49	45.4	77	47.5	0.727
Add bleach/chlorine	26	22.8	12	24	14	21.9	0.788	64	23.7	27	25	37	22.8	0.683
Others^##^	4	3.5	3	6	1	1.6	0.201	14	5.2	9	8.3	5	3.1	0.057

* P<0.05 significant by *χ*^2^ test

# Responses were yes and no, yes response is presented

## include decantation, solar disinfection, using water filter e.g. sand, ceramic etc. and straining using cloth

Most of the mothers/caregivers (children aged 6-11 months = 52.6%; children aged 12-23 months = 60.4%) stored water in a clean and covered container.

### Personal hygiene practices among mothers/caregivers Rongai sub-county

Personal hygiene practices among mothers/caregivers in the two child age categories and agro-ecological zones are presented in Table 3. Washing hands in a shared bowl of water was the common hand washing practice among mothers/caregivers (children aged 6-11 months = 28.1%; children aged 12-23 months = 28.5%). Many mothers/caregivers (P<0.05) from high potential areas (children aged 6-11 months = 92.2%; children aged 12-23 months = 80.2%) washed hands after cleaning their baby's bottom compared to those from low potential areas (children aged 6-11 months = 74%; children aged 12-23 months = 69.4%). There was a significant difference (P<0.05) between mothers/caregivers of children aged 12-23 months in the low potential and high potential areas who washed their hands after going to the toilet/latrine (Low Potential areas=20.4%; High Potential areas =9.3%) and covered food to avoid contamination (Low Potential areas=20.4%; High Potential areas=9.3%) compared to their counterparts in areas.

**Table 3:** Personal hygiene practices among mothers/caregivers Rongai sub-county

	Children aged 6 – 11 months							Children aged 12 - 23 months						

	Total		Low potential area		High potential area		P value	Total		Low potential area		High potential area		P value

	n	%	n	%	n	%		n	%	n	%	n	%	
Washes hands in shared bowl of water	32	28.1	16	32	16	25	0.409	77	28.5	34	31.5	43	26.5	0.379
Someone pouring clean water from a jug onto one's hands	16	14	8	16	8	12.5	0.593	46	17.1	12	11.1	34	21.1	0.033*
Washes hands under running water	13	11.4	5	10	8	12.5	0.677	39	14.4	13	12	26	16	0.358
Washes hands with soap	49	43	19	38	30	46.9	0.342	97	36.1	50	46.3	47	29.2	0.004*
Washes hands after cleaning the baby's bottom	96	84.2	37	74	59	92.2	0.008*	205	75.9	75	69.4	130	80.2	0.042*
Washes hands after going to the toilet/latrine	8	7	4	8	4	6.2	0.717	37	13.7	22	20.4	15	9.3	0.009*
Don't know how to avoid faecal contamination	108	94.7	47	94	61	95.3	0.755	260	96.3	106	98.1	154	95.1	0.188
Washes hands before preparing/handling food	91	79.8	42	84	49	76.6	0.326	226	83.7	96	88.9	130	80.2	0.060
Washes hands before feeding a child/eating	84	73.7	36	72	48	75	0.718	188	69.6	76	70.4	112	69.5	0.829
Washes hands after handling raw food	63	55.3	23	46	40	62.5	0.790	171	63.3	66	61.1	105	64.8	0.536
Washes hands after handling garbage	74	64.9	33	66	41	64.1	0.830	189	70	71	65.7	118	72.8	0.212
Washes hands after shaking other peoples' hands	37	32.5	20	40	17	26.6	0.128	87	32.2	39	36.1	48	29.6	0.264
Removes faeces from the home and surroundings	42	36.8	18	36	24	37.5	0.869	109	40.4	50	46.3	59	36.4	0.105
Covers food to avoid faecal contamination	8	7	4	8	4	6.2	0.717	37	13.7	22	20.4	15	9.3	0.009*
Composite personal hygiene practices indicator (adequate practices)	46	40.4	15	30	31	48.4	0.046*	102	37.8	39	36.1	63	38.9	0.645

* P<0.05 significant by *χ*^2^ test

### Health characteristics of children aged 6-23 months

Table 4 presents a comparison of health characteristics of children aged 6-11 and 12-23 months of Rongai subcounty across the two agro-ecological zones. The health characteristics were for the previous two weeks duration based on the mothers/caregivers report. Overall, many children were reported to have experienced fever (children aged 6-11 months = 61.4%; children aged 12-23 months = 60%). More children aged 12-23 months from high potential areas (64.8%) experienced fever as compared to those from low potential areas (52.8%). Overall, about a third of the children experienced diarrhoea (children aged 6-11 months = 29.8%; children aged 12-23 months = 31.5%). All children aged 6-11 months from low potential areas (100%) had been diagnosed with parasites while 9 out of 10 children were reported in high potential areas. Majority of children aged 6-11 months (93%) and aged 12-23 months (74%) had been treated for parasites in the previous 6 months Nutrition Outcomes of Children aged 6-23 months in Rongai sub-county

**Table 4:** Child health characteristics within the two age groups of children in Rongai sub-county

	Children aged 6 – 11 months							Children aged 12 - 23 months						

	Total		Low potential area		High potential area		P value	Total		Low potential area		High potential area		P value

History of illness in the previous 2 weeks	n	%	n	%	n	%		n	%	n	%	n	%	
Fever	70	61.4	29	58	41	64.1	0.509	162	60	57	52.8	105	64.8	0.048*
Cough or cold	34	29.8	13	26	21	32.8	0.430	83	30.7	28	25.9	55	34	0.162
Fast breathing or shortness of breath	104	91.2	46	92	58	90.6	0.797	242	89.6	92	85.2	150	92.6	0.050
Diarrhoea	34	29.8	16	32	18	28.1	0.654	85	31.5	43	39.8	42	25.9	0.016*
Diagnosed with parasites	106	93	50	100	56	87.5	0.010*	240	88.9	98	90.7	142	87.7	0.429
Treated for parasites in the last 6 months	106	93	48	96	58	90.6	0.265	202	74.8	76	70.4	126	77.8	0.170

* P value<0.05 significant by *χ*^2^ test

**Figure 1:**
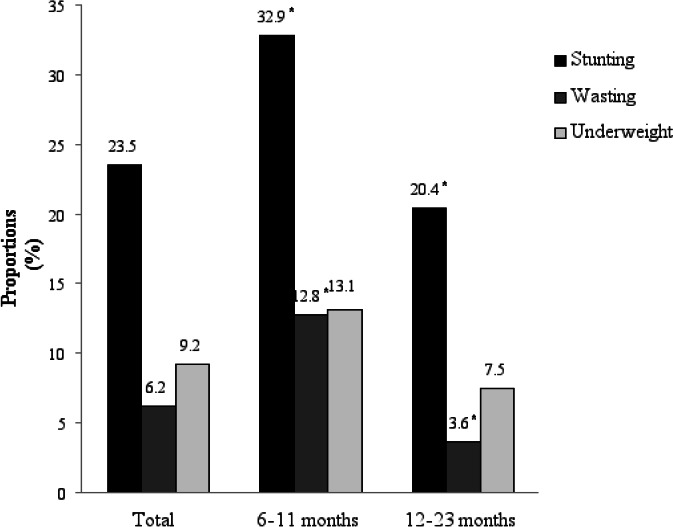
Z-scores of children aged 6-23 months across the two age categories of 6-11 months and 12-23 months (*P<0.05)

Figure 1 presents the prevalence of under-nutrition among children aged 6-23 months of Rongai sub-county compared across two age categories of 6-11 and 12-23 months. The prevalence of stunted children was 23.5% while wasted and underweight children were 6.2% and 9.2% respectively. Of the stunted and wasted children, a higher proportion (P>0.05) were in the 6-11 month age category compared to those aged 12-23 months.

### Hand washing and Hygiene factors associated with diarrhoea among children

Table 5 shows the hygiene factors that were associated with diarrhoea in children aged 6-11 months. The practice of washing hands under running water [AOR=0.078 95% C.I; (0.008±0.795)], washing hands before feeding the child [AOR=0.175 95% C.I; (0.043±0.709)] and after handling garbage [AOR= 0.273 95% C.I; (0.089±0.831)] reduced the chances of experiencing diarrhoea among children. In children aged 12-23 months, none of the hygiene factors was significantly associated with childhood diarrhoea (P>0.05).

**Table 5:** Hand washing and Hygiene factors associated with diarrhoea among children aged 6-11 months of Rongai sub-county

	B	AOR	95% C.I.for AOR	P value
Pouring clean water from a jug onto one's hands	-0.268	0.765	0.191±3.054	0.704
Washes hands under running water	-2.545	0.078	0.008±0.795	0.031*
Washes hands with soap or ashes	0.719	2.053	0.707±5.967	0.186
Washes hands after cleaning the baby's bottom	0.577	1.781	0.304±10.419	0.522
Removes faeces from surrounding	0.030	1.030	0.348±3.050	0.957
Don't know how to avoid faecal contamination	0.054	1.055	0.093±12.032	0.965
Washes hands after going to the toilet	0.647	1.909	0.182±20.010	0.590
Washes hands before handling food	0.427	1.533	0.426±5.521	0.513
Washes hands before feeding child	-1.741	0.175	0.043±0.709	0.015*
Washes hands after handling raw foods	-0.303	0.739	0.259±2.105	0.571
Washes hands after handling garbage	-1.300	0.273	0.089±.831	0.022*
Treats water to be safe	0.510	1.666	0.548±5.061	0.368
Boils water for safety	0.503	1.654	0.589±4.645	0.339
Adds bleach for water safety	0.587	1.799	0.606±5.340	0.290
Strain water	-1.126	0.324	0.021±5.015	0.420
Don't know how to treat water for safety	-1.184	0.306	0.029±3.212	0.324
Composite hygiene practices indicator (adequate practices)	-1.030	0.357	0.046±2.767	0.324

* P<0.05 significant using binary logistic regression; B-estimated coefficient; AOR-adjusted odds ratio; C.I-confidence interval

### Childhood illnesses associated with under-nutrition of children in Rongai sub-county

The childhood illnesses associated with under-nutrition across the two age groups are presented in Table 6. Having trouble breathing [AOR=6.212 95% C.I; 1.596±24.184] and being treated for parasites [AOR=3.123 95% C.I; 1.047±9.317] were the only illnesses that were positively (P<0.05) associated with underweight among children aged 6-11 months. There were no associations (P>0.05) between child illnesses and being stunted or wasted. None of the illnesses was significantly associated with underweight, wasting or stunting among children aged 12-23 months.

**Table 6:** Childhood illnesses associated with malnutrition of children in Rongai sub-county across the 6-11 and 12-23 month age groups

	B	AOR	95% C.I.for EXP(B) Underweight	P Value
Fever	0.465	1.592	0.450±5.635	0.471
Cough or cold	-1.133	0.322	0.097±1.074	0.065
Fast breathing/shortness of breath	1.826	6.212	1.596±24.184	0.008*
Diarrhoea	0.569	1.766	0.508±6.140	0.371
Treated for parasites	1.139	3.123	1.047±9.317	0.041*
	**Wasting**			

Fever	0.496	1.642	0.303±8.910	0.565
Cough or cold	-0.132	0.877	0.147±5.219	0.885
Diarrhoea	1.341	3.824	0.412±35.495	0.238
Treated for parasites	0.350	1.419	0.252±7.996	0.692
	**Stunting**			

Fever	-0.096	0.908	0.450±1.833	0.789
Fast breathing/shortness of breath	-0.277	0.758	0.234±2.456	0.644
Diagnosed with parasites	-1.133	0.322	0.067±1.558	0.159
Treated for parasites	-0.297	0.743	0.316±1.748	0.496

* P<0.05 significant using binary logistic regression; B – Regression co-efficient; AOR – Adjusted Odds Ratio; C.I – Confidence Interval

## Discussion

The main aim of this study was to assess the hygiene practices of mothers/caregivers of children aged 6-23 months and how these factors are associated with childhood illnesses and subsequently under-nutrition. Access to water that is safe for drinking is considered a basic human right and is important for good health as per the WHO guidelines on drinking water quality^18^. Boreholes and surface water were cited as the most common sources of water for households in Rongai sub-county. Water sourced from unimproved sources such as surface water, has an increased risk of illnesses and spread of water-borne diseases especially among young children^7^. The proportion of households (20.2%) that sourced surface water was similar to national trend (24%)^7^. Rainwater was the least common water source at the time of the study. This is because the study was carried out when there was minimal rainfall in the area. It is common for water supplies to be contaminated by pathogens from the surrounding environment^19^. Therefore, it is imperative that proper treatment and storage of drinking water at home is observed as recommended by the WHO water safety plan where household water treatment and storage is seen as an effective measure to improve the quality of drinking water^20^. In Rongai sub-county, many of the households used soap and water as a method of treating containers used for fetching and storing water, which is considered as an appropriate practice. However, a large number of the households did not treat their water containers at all yet water treatment and safe storage has been associated with reduced chances of water-borne infections^20^,^21^. Most of the households (54.9%) reported to be treating their drinking water before consumption, which was slightly higher than the national trend of 46%^7^. This proportion is even higher than the documented 18.2% of populations in Africa who treat their water at household level^22^,^23^. The most common method of water treatment was boiling at 49.2% compared to 24% nationally^7^. This was mainly because boiling water for household consumption is an inexpensive and easy to do method of water treatment as it does not require any special equipment or chemicals and the fuel used (firewood) is readily available in the study area. Moreover, studies have recommended boiling water as the preferred method of treating household water especially in emergency situations^24^

A high percentage of the children reported to have experienced fast breathing/shortness of breath, an indicator of acute respiratory tract infections, over the previous two weeks. Prevalence of fever in Rongai sub-county was 30.2% among age group 6-11 months, which was similar to the national trend of 31.2%^7^. This could indicate compromised immunity among these children attributable to inadequate nutrition as observed in other studies^20^,^25^,^26^. Reported cases of fever were very high among the 12-23 months age group at 69.8% with national prevalence being 29.9%^7^. This high prevalence could be due to these children being considered old enough not to be easily susceptible to such infections hence mothers/caregivers may fail to pay attentive care to them. Fever and diarrhoea among children have been known to be associated with under-nutrition hence resulting in growth failure, impaired cognitive development and reduced immunity^27^-^30^. Over 1 million deaths among children are caused by diarrhoea annually contributing to the global burden of disease^31^,^32^. In Kenya, the prevalence of diarrhoea is highest among children aged 6-11 months and 12 – 23 months at 27% and 24% respectively^7^. In this study, the prevalence was at 29.8% for children aged 6-23 months and 31.5% for those aged 12-23 months^7^. Studies have documented 38% of deaths among children under five years to be caused by diarrhoeal infections^33^ which have been linked to poor hygiene practices^32^. Washing hands under running water, before feeding child and after handling garbage were some of the practices that were significantly associated with reduced chances of getting diarrhoea among children aged 6-11 months in this study. This is similar to observation and intervention studies where proper hand-washing practices were associated with significantly reduced chances of diarrhoea^34^-^36^. Hygiene practices during preparation, feeding and storage of complementary feeds is important since Verdeja et al (2019)^37^, examined associations among childhood disease and various WASH conditions and behaviour in rural Tanzania and concluded significant association between improved WASH practices and reduced risk of childhood illnesses. Adhering to appropriate hand-washing practices is therefore important in reducing chances of children getting diarrhoea^8^,^45^,^47^ as reported in a study done in Ethiopia where washing hands during the critical times was associated with reduced risk of diarrhoeal cases since contaminated complementary feeds are a source of transimssion^38^,^48^.

The childhood illnesses reported by the mother/caregiver did not have significant associations with stunting or wasting despite other studies reporting associations between infectious diseases and stunting^39^-^40^. However, fast breathing/shortness of breath and receiving treatment for parasites increased the chances of a child aged 6-11 months being underweight. This concurs with a study in Nigeria where child illnesses were considered to predict chances of a child being underweight thus maintaining that disease and illnesses affect nutritional status^41^. Furthermore, under-nutrition among children has been associated with poor hand-washing practices by their mothers/caregivers^42^-^44^, ^48^. This corroborates the established hygiene, infection and under-nutrition synergy as documented previously in various studies^4^,^9^,^10^.

## Conclusion

Washing hands using the appropriate procedures and at the critical times is important in reducing risks of diarrhoea. Mothers/caregivers in Rongai sub-county exhibited proper practices on some of the key hand-washing times and appropriate hand washing procedures, however, many of them did not practice proper sanitation regarding handling of faecal waste in order to avoid contamination. It is important to educate caregivers of young children about the importance of being keen on the critical hand washing times during complementary feeding period. This will reduce incidences of diarrhoea and parasitic infections and subsequently improved nutritional outcomes. Behaviour change communication targeting mothers/caregivers of children aged 6-11 months could be considered as an intervention to improve hygiene practices, reduce risof childhood infections/illnesses and alleviate long term effects of under nutrition. These BCC strategies may include conducting periodic health talks and demonstrations on proper hygiene practices to increase awareness; develop cost-effective handwashing devices near latrines to encourage handwashing and making of home visits by health care workers to promote proper practices.
